# Strengthening policies and structures to combat illicit tobacco trade in the Philippines

**DOI:** 10.3389/fpubh.2023.1089853

**Published:** 2023-01-25

**Authors:** John Rafael Y. Arda, Alen Josef A. Santiago

**Affiliations:** School of Government, Ateneo de Manila University, Quezon City, Philippines

**Keywords:** tobacco, Philippines, policy, government, law enforcement, illicit trade

## Abstract

The Philippines has been seeing an increase in illicit tobacco trade in recent years, undermining the impacts of legal measures such as tobacco products' taxation and regulation due to circumvention of established avenues and costing the government its revenue. Currently, the country has twelve policies related to the prevention of illicit tobacco trade with gaps identified in its lack of licensing systems for tobacco retailers and policies on law enforcement cooperation, which manifests in the country being fully compliant to only 5 of the 16 articles under the World Health Organization's Illicit Tobacco Trade Protocol. It is recommended that the country establish a national agency or framework specifically for illicit tobacco trade to address its gaps under Tracking and Tracing, Due Diligence, and Unlawful Conduct.

## Introduction

Illicit tobacco trade is an issue that the country is currently facing. Based on the prevalence of smoking from the National Nutrition Health Survey and the prevalence of cigarette consumption from the Global Adult Tobacco Survey, a gap analysis done against the legal sales revealed that the share of illicit tobacco trade was initially decreasing from 1998 to 2013, then increased continuously up until 2018 (1). The market size of illicit tobacco is estimated to have been at 16% in 2018, costing the government millions in revenue (1). With illicit tobacco, legal measures such as taxation on tobacco products and its accessibility are impacted due to circumvention of its established avenues (2). Thus, not only does it impact the average price of these commodities, but it also increases disparity in its use among income populations, the choice of brands, and the age group that can access it, and thus, experience its health consequences.

The World Bank estimates that 1.1 billion people smoke globally, which accounts for 21% of the world's adult population (2). Smoking kills half of the long-term smokers, accounting for more deaths each year than HIV/AIDS, tuberculosis, and malaria combined at around 7.2 million people yearly. This results in a worldwide economic cost of around US$1.4 trillion per year, equivalent to 1.8% of the world's Gross Domestic Product. Around 40% of these costs are incurred in developing countries, a testament to how the burden of smoking is disproportionately distributed across nations (2).

The global share of the illicit cigarette market is 11.6%, equivalent to 657 billion cigarette sticks a year and a loss of $40.5 billion in revenue (3). If it were eliminated, governments would gain at least $31 billion, and from 2030 onwards would save over 160,000 lives a year. Just like tobacco use, however, the burden of the illicit trade falls mainly on lower income countries, with its share being 16.8% of their cigarette market on average. This is compared to the 9.8% average in high income countries. Thus, being able to remedy this problem would result in a net gain that's proportionately higher for these lower income countries, an estimated $18.3 billion in revenues.

The Philippines is the second ranked country in the Southeast Asia region in terms of number of smokers (4). 23.8% Filipino adults and 12% of the youth population smoke tobacco (5). This has resulted in 87,600 Filipinos deaths from tobacco-related diseases annually, costing the country PHP 188 billion (USD 8 billion) yearly from expenditures on healthcare, income foregone, and premature deaths (6). Moreover, two of the four diseases are also part of the top three overall causes of death locally, with ischemic heart diseases being the largest contributor while cancer being the third. For cancers, lung cancer is the second largest cause of death, just behind breast cancer (7).

As a result of illicit tobacco, the consequences of tobacco smoking disproportionately affect those who are more likely to have access to illicit supplies of the product, specifically those of a lower income population. This incurs greater cost due to the increased consumption of cigarettes despite the presence of anti-tobacco policies, which undermines efforts the government has done to decrease the prevalence of tobacco smoking, hence the need to combat illicit tobacco effectively if the country wants to implement anti-tobacco legislation as effectively as possible (2).

## Policy options and implications

At the governance level, the Philippines has 12 policies related to the prevention of illicit tobacco trade (five republic acts, three implementing guidelines, two revenue regulations, a memorandum circular, and a presidential decree) (8). Table 1 contains descriptions of each policy and what it corresponds to. In terms of the Worth Health Organization's Illicit Tobacco Trade Protocol, the country is fully compliant to only five of the 16 articles, while partially compliant to four more. The five articles the country is compliant to are Free Zones and International Transit, Liability of Legal Persons, Seizure Payments, Special Investigative Techniques, and Protection of Personal Data. Partial compliance has been achieved for four more articles, namely Licensing, Recordkeeping, Duty-free Sales, and Prosecutions and Sanctions. The seven articles the Philippines is non-compliant to are Tracking and Tracing, Due Diligence, Unlawful Conduct, Security and Preventive Measures, Sales by Internet or Other Technology, Disposal or Destruction, and General Obligation (8, 9).

**Table 1:** Laws governing illicit tobacco trade in the Philippines.

**Policy**	**Agency-in-charge**	**Description**
Tax Reform Act of 1997 (RA 8424)	Bureau of Internal Revenue	An act rationalizing the Philippine internal revenue tax system, including tax administration.
Electronic Commerce Act of 2000 (RA 8792)	Department of Trade and Industry	An act providing for the recognition and use of electronic commercial and non-commercial transactions.
Civil Service Commission—Department of Health—Joint Memorandum Circular 2010-01	Civil Service Commission—Department of Health	Provides limitations to relationships of government agencies with the tobacco industry.
Implementing Rules and Regulation—Food and Drug Administration (FDA) Act of 2009 (RA 9711)	Department of Health—Food and Drug Administration	Rules and regulations implementing The Food and Drug Administration Act of 2009 (RA 9711).
Sin Tax Law (RA 10351)	Bureau of Internal Revenue	An act amending the National Internal Revenue Code of 1997, restructuring the excise tax on alcohol and tobacco products, as amended by RA 9334.
Tobacco Tax Law of 2019 (RA 11346)	Bureau of Internal Revenue	An act amending the National Internal Revenue Code of 1997, increasing excise tax on tobacco products and imposing taxes on excise tax on heated products and e-cigarettes, as amended by RA 10963 or the Tax Reform for Acceleration and Inclusion (TRAIN) in 2017.
Data Privacy Act of 2012 (RA 10173)	National Privacy Commission	An act protecting individual personal information in information and communications systems in the government and the private sector.
Tariff and Customs Code of the Philippines [RA 1937 (1957), amended in RA 4172 (1966), and consolidated in the Presidential Decree 1464 (1978)]	Department of Finance	There are other general policies on trade and tariffs in the Philippines that govern the overall accountability, implementation, finance, and monitoring of traded goods.
2010 Amended Rules and Regulations Governing the Trading and Redrying of Locally Grown Leaf	National Tobacco Administration	Implementing guidelines governing the trading of locally grown leaf tobacco.
Amended Rules and Regulations Governing the Exportation and Importation of Leaf Tobacco and Tobacco Products	National Tobacco Administration	Implementing guidelines in facilitating the processing of exportation and importation of leaf tobacco and tobacco products.
Revenue Regulations No. 7-2014	Bureau of Internal Revenue	Prescribes the affixture of new internal revenue stamps on imported and locally manufactured cigarettes, whether for domestic sale or for export, and the use of the Internal Revenue Stamp Integrated System (IRSIS) for ordering, distribution, and monitoring.
Revenue Regulations No. 17-2012	Bureau of Internal Revenue	Prescribes the Implementing Guidelines on the revised tax rates on alcohol and tobacco products pursuant to the provisions of RA 10351 and clarifies certain provisions of existing Revenue Regulations.

Policy gaps identified are the lack of licensing systems for tobacco retailers and policies on law enforcement cooperation. No national policies mandate licenses being mandatory for retailers, even though it is stated in Article 6 of the Illicit Tobacco Trade Protocol provisions (8). This makes information needed for tracking tobacco dealers unavailable for agencies such as the Bureau of Internal Revenue or Bureau of Customs to use in combatting illicit tobacco. These agencies are also dependent on higher authorities when it comes to implementation, subsuming policies, and strategies in place under national efforts. It is recommended that the country establish a national agency or framework for illicit tobacco trade specifically to address these gaps.

At the level of financing, tobacco illicit trade is shown to result in revenue loss for the Philippines (1, 10). A comparison between cigarette legal sales and estimated cigarette consumption from 1998 to 2018 in the Philippines, revealed that “legal sales initially increased from 70 billion sticks in 1998 to its peak in 2013 with 97 billion sticks” (1). Moreover, the current Sin Tax Reform can still be improved on due to issues in implementation and success in changing administrations (11). Regardless, its impact has been significant, tripling the government revenue from PHP32.9B to PHP106B, which resulted in a national health budget more than double the initial, covering 8 million more low-income families under the National Health Insurance Program (12).

At the delivery level, the Bureau of Internal Revenue has implemented the Internal Revenue Stamp Integrated System, a strategy that identifies whether a cigarette pack has paid excise tax properly and has been examined by the Bureau of Customs before its delivery to the market (13, 14). The compliance rate as of May 2016 was around 95.8% (12). An alleged use of counterfeit stamps in 2017 was also reported and dealt with by the appropriate agencies (15). Despite this, Lavares et. al (1) still argued that there is not enough mechanism to monitor delivery of illicit tobacco trades as well as measuring “the trend in the gap between tobacco consumptions and legal sales”.

## Actionable recommendations

To address gaps brought up by the latest policy review by Geroy and Encarnacion (8), recommendations were structured around addressing the lack of a national coordinating body for the various agencies that handle illicit tobacco-related tasks thus improving compliance to the Illicit Tobacco Trade Protocol articles that the country is behind in, particularly Tracking and Tracing, Unlawful Conduct, and Due Diligence. Recommendations were made through using related words to search for literature on national implementing agencies and measures against illicit tobacco trade in Google Scholar and PubMed up until the fifth page, after which a different keyword or combination of keywords were entered to ensure ample coverage of related studies. The results were then put together as actionable recommendations.

**Figure 1:**
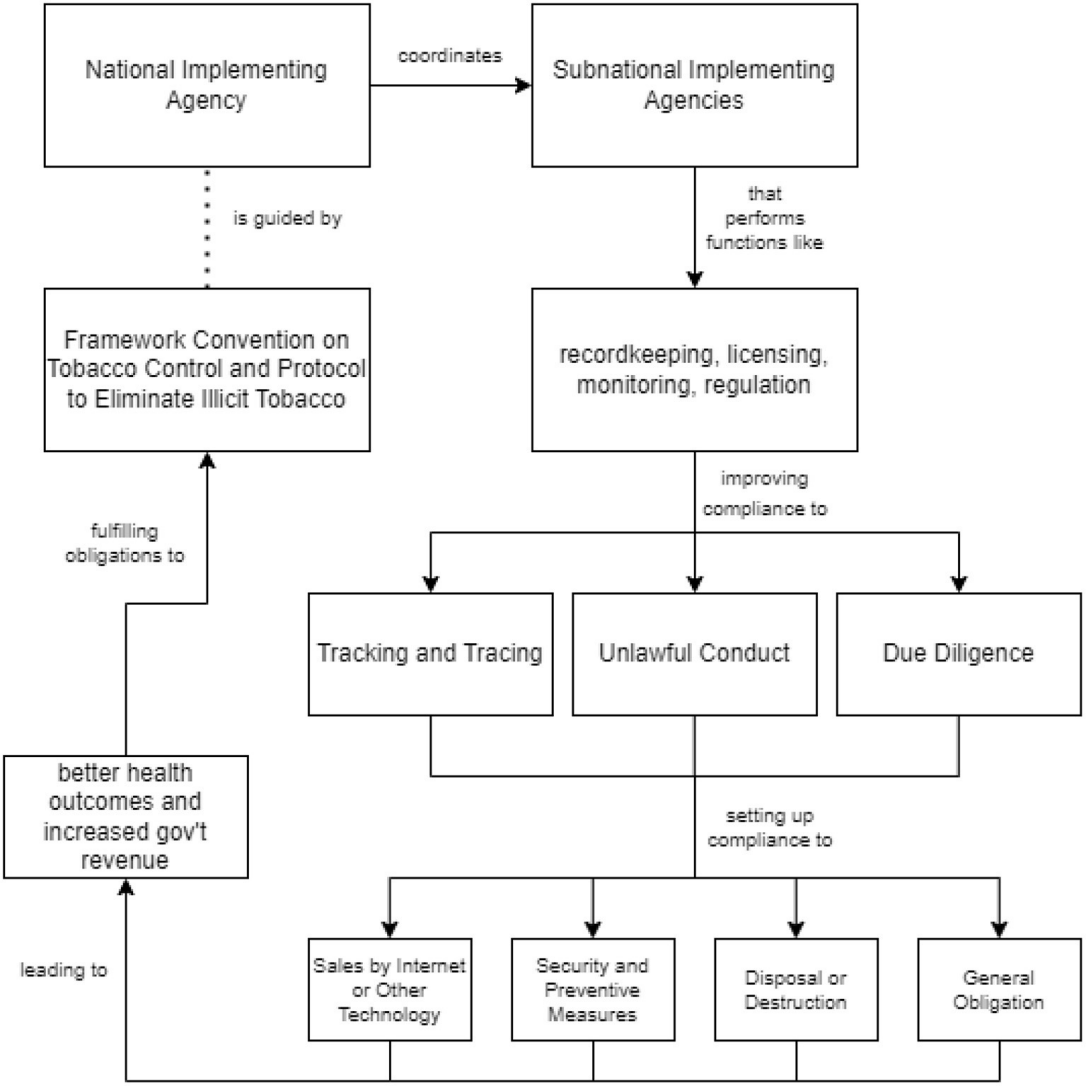
Conceptual framework.

The approaches described allow the government to address the issue of illicit tobacco on multiple levels, as well as implement measures that cover gaps in its framework prior to adapting the approaches. By establishing a national implementing agency that can monitor collaboration between involved agencies, the country's fulfillment of provisions in the Illicit Tobacco Trade Protocol can be increased and thus, improve both government revenue and public health. This is summarized in the flowchart depicted in Figure 1.

The first is to assign a national implementing agency or a regulatory agency through a law or mandate for monitoring the illicit tobacco trade activities in the Philippines. Establishing national agencies and frameworks allows counties to make better national-level decisions to facilitate implementation of protocols both within the country and when working with other signatory countries of the Framework Convention on Tobacco Control (16). They are also beneficial in addressing costs and overall quality of programs, as well as in implementing programs that deal with improvement of health outcomes (17–19).

National agencies and frameworks are also central in establishing a culture of collaboration among agencies involved in the implementation, policy, and regulation of illicit tobacco trade. For efforts against illicit tobacco trade, it is important to develop local and regional partnerships between tax authorities, customs, police, retail inspectors, and the public health community to enhance feasibility, effectiveness, and likelihood of achieving long-term results (20). The adoption of a national agency or framework that can streamline collaborations among agencies involved in relevant programs' implementations can maximize the benefit of collaboration in government efforts, such as increased effectiveness of program outcomes, shared responsibility, and management for goals, and flowing and available relevant information (8, 21–24). For illicit tobacco trade specifically, having timely information available is important due to how dynamic and central it is to related programs' implementation (21).

To improve the overall situation of illegal tobacco trade policies in the country, the second recommendation focuses on filling in the gaps identified in current policy according to the Illicit Tobacco Trade Protocol, such as Tracking and Tracing, Due Diligence, and Unlawful Conduct. It is known that through the continued adoption of methods outlined in the Illicit Tobacco Trade Protocol by countries, it can improve capacities to reduce illicit tobacco products globally, hence improving public health (20). Having a proper track and tracing system can prevent a country's involvement in illicit trade schemes such as in illicit tobacco, which is why addressing tracking and tracing through the inclusion of retailers into the licensing system for selling tobacco-related products is one of the steps necessary to take in the implementation of Illicit Tobacco Trade Protocol, as they can be seen as intermediaries at times between the tobacco product suppliers and the consumers (16, 20). This function as an intermediary is important to monitor due to its risk of becoming exploitable by illicit trade, which is why requiring licenses can expand the measures available for use by the government to reduce the risk of participating in it through its ability to place restrictions on the licensee if there is a perceived threat (16). It is also highly feasible, with estimates finding that the fees licensing systems impose can cover the costs that come with implementing it, such as the staff involved, the equipment to be used, and the development of the application itself (25).

Licensing systems can also help in implementing a central registry, which minimizes confusion and slow responses in anti-illicit tobacco related efforts (20). Along with imposing license requirements on retailers, better use of the stamp verification process develops a robust tracking and tracing system along with diligence among retailers and suppliers, minimizing unlawful conduct (12, 26). With a proper monitoring and evaluation process in place, along with adjustable anti-tobacco trade measures, a gross return of around £10 per £1 invested is yielded, which is estimated to be around PHP671 per PHP67 (25). This is important as it helps combat the tobacco industry's economic arguments against further implementation of these measures, with their influence over policy being grounded on perceived economic losses across multiple countries' governments (27–32). Overall, the cost of implementing Illicit Tobacco Trade Protocol, should a national system as what was described in the first recommendation is established, is estimated to be around $14M–$83M annually, which is <10% of the estimated gain should illicit tobacco be dealt with through the proper implementation of measures laid out in the protocol (33).

Before the successful implementation of these recommendations, related barriers to them need to be addressed beforehand. In the patient level, issue of the sale of counterfeit tobacco products from other countries being accessible to smokers online is a concern that needs to be addressed due to its potential to circumvent currently existing laws (16). It is imperative then that regulation on or strengthening of the monitoring of illegal tobacco products on the internet or online shopping sites is adapted along with them (34). For the professional level, failure of printing, media, and advertising professionals to adhere to existing regulations, coupled with the prevalence of tobacco use among health care workers, make it hard to promote smoking cessation among the public (35). Stricter implementation of Republic Act No. 9211, the Tobacco Regulation Act of 2003, will be necessary in making sure smoking is limited to designated areas around workspaces (35). For the organizational level, there is a perceived lack of understanding with relevant laws such as excise cigarette tax or a common framework to combat illicit tobacco trade (2, 8). Providing training that capacitates members of regulatory bodies to implement frameworks to combat illicit tobacco trade can help combat this barrier along with the possible resistance coming from the tobacco industry, which will use arguments grounded on misinterpretations such as increased cigarette smuggling being a result of anti-tobacco policies (8, 36, 37). Lastly, systematic interference from the tobacco industry in policy development is one of the most prevalent barriers to anti-tobacco efforts, which is why it will become necessary going forward for efforts against illicit tobacco trade to be supplemented by existing laws that prohibit the interference of the tobacco industry with anti-tobacco endeavors through collaboration with groups that monitor and counter tobacco industry interference (38–40).

## Conclusion

The Philippines is currently lacking in policies that address illicit tobacco trade as evaluated under the Illicit Tobacco Trade Protocol, being compliant to only 5 of its 16 articles. To address gaps in policy and estimated revenue losses from illicit trade, it is recommended that the country establish a national agency or framework that focuses on efforts against the issue specifically, as having a more specialized entity on the matter opens options to improve overall efficiency, quality, and collaboration among concerned agencies across multiple sectors. In doing this, actions such as retail licensing and improvement of the stamp verification process will become easier to implement nationwide and improve overall compliance to the Illicit Tobacco Trade Protocol.

However, there are challenges to consider in terms of implementing these recommendations. The shift to online platforms of illegal tobacco trade, the prevalence of tobacco use among healthcare workers, proper training for members of regulatory bodies, and reinforcing of existing laws against tobacco industry interference are some of the factors to consider when acting upon the recommendations detailed within the paper.

## Author contributions

JA led the writing of the policy brief. AS compiled relevant policies and handled stakeholder-related logistics for further input and feedback. Both authors contributed to the making of the manuscript draft.
